# ANTI-FUNGAL ACTIVITY OF *ACALYPHA WILKESIANA*: A PRELIMINARY STUDY OF FUNGAL ISOLATES OF CLINICAL SIGNIFICANCE

**DOI:** 10.21010/Ajid.v16i1.4

**Published:** 2021-12-21

**Authors:** Katibi Oludolapo Sherifat, Aboh Mercy Itohan, Salawu Oluwakayinsola Adeola, Kola-Mustapha Adeola, Olatunji Lawrence Aderemi

**Affiliations:** 1 Dermatology Unit, Department of Paediatrics and Child health, University of Ilorin, Ilorin, Nigeria.; 2 Department of Microbiology and Biotechnology, National Institute for Pharmaceutical Research & Development, Abuja, Nigeria.; 3 Department of Pharmacology & Toxicology, National Institute for Pharmaceutical Research & Development, Abuja, Nigeria; 4 Department of Pharmaceutics and Pharmaceutical Microbiology, Faculty of Pharmaceutical sciences, University of Ilorin, Ilorin, Nigeria; 5 Department of Physiology, College of health Sciences, University of Ilorin, Ilorin, Nigeria

**Keywords:** Anti-fungal, *Acalypha wilkesiana*, skin diseases, ethnobotanical medicine

## Abstract

**Background:**

*Acalypha wilkesiana* (AW, a popular medicinal plant has been used in traditional medicine to treat a variety of skin disorders including pityriasis versicolor and seborrheic dermatitis. As a prelude to clinical trials in humans, an experimental study was carried out to determine the spectrum of antifungal activity of 2 variants of the *Acalypha wilkesiana* plant.

**Materials and Methods:**

The ethanol extract and herbal cream formulation of the dried leaves of 2 cultivars (Macrophylla & Hoffmani) of *Acalypha wilkesiana* were investigated for *in-vitro* antifungal activity by disc diffusion and micro-broth dilution techniques. Organisms tested were typed cultures of *Malassezia furfur*, *Candida albicans* and *Trichophyton rubrum*; and clinical strains of *Microsporum canis* and *Epidermophyton floccosum*.

**Results:**

Both cultivars (Macrophylla and Hoffmanii) of the plant showed good activity against all the fungi tested except *Microsporum canis* (8.0±0.00; 7.00±0.00 mm). The greatest activity was observed against *Trichophyton rubrum* (22.0±0.00; 24.00±0.00 mm). The Minimum Inhibitory Concentration (MIC) of the crude extract ranged between 0.25 and 8 mg/ml for all organisms, while that of the herbal cream was 0.31-8mg/ml. The lowest MIC was seen with *Candida albicans* for both varieties of the plant. The *Acalypha wilkesiana* Hoffmanii demonstrated a greater activity against *Candida albicans* and *Malassezia furufur* than the *A. wilkesiana* Macrophylla.

**Conclusion:**

This study reveals *Acalypha wilkesiana leaf extract* has potential for development as a cream that can be used to treat superficial fungal skin infections.

## Introduction

*Pityriasis versicolor* (PV) disease is a chronic, superficial fungal infection of the skin caused by the lipophilic, yeast-like fungus, *Malassezia*. This organism formerly known as *Pityrosporum* is a saprophytic yeast that is part of the normal skin flora. There are about 14 species identified of which *Malassezia furfur* is the commonest found in Nigeria. Common names for PV are *Tinea flava*, *Dermatomycosis furfuracea* and *Tinea versicolor* (Sharma *et al.*, 2011).

In recent years, there has been a considerable renewal of interest in the use of medicinal plants in developing countries because herbal medicines have been reported so be safe, easily accessible and without any adverse side effect especially when compared with orthodox drugs. 2 The search for antimicrobial agents from plants like garlic, ginger, thyme, tea leaves have been a growing interest in the last few decades. Results generated from many of these studies cannot, however, be directly compared due to the absence of standardization in particular antimicrobial methods employed (Kunle *et al.*, 2012). Many researchers have proposed the need for established methods with consistent results for the evaluation of antimicrobial activities from plant extracts (Iniaghe *et al.*, 2009). Superficial fungal skin disease is a common health problem particularly in tropical countries like Nigeria. With the constant challenge of discovering new drugs to replace existing ones due to antimicrobial resistance, preliminary studies on their efficacy are needed to verify claims of clinical efficacy in humans. *Acalypha wilkesiana*, a popular medicinal plant has been used by herbal doctors to treat PV, a common superficial mycosis that is associated with cosmetic disfigurement and reduced quality of life. Experimental studies have demonstrated the significant activity of this plant against various fungi. However, few studies have studied extensively the antifungal activity of *Acalypha wilkesiana* against *Malassezia furfur*, the aetiological organism of *P. versicolor*.

*Acalypha wilkesiana* Muell Arg., commonly known as copper leaf, is a plant from the family *Euphorbiaceae* (spurge family). The Leaf poultice have been used for headache, swelling, cold and wound dressing. Chopped pieces of the dried stem and root in past studies were steeped in alcohol and used for stomach ache and as worm expellant in man in the Delta region of Nigeria (Onocha and Olusanya, 2010). The leaves of this plant are eaten as vegetables in the management of hypertension, in Southern Nigeria (Iwu, 1993).

Past studies have documented the phytochemical constituents of *Acalypha wilkesiana*. A comparative evaluation of the antimicrobial activities and phytochemical screening of two varieties of AW showed that the inhibitory effect against microorganisms differ among the varieties of this plant (Oladunmoye, 2006).

Extracts of the leaves of *Acalypha wilkesiana* (Macrophylla) have been shown to possess a wide range of antibacterial and antifungal activity (Oladunmoye, 2006; Alade and Irobi, 1993; Adeshina *et al.*, 2010). Oyelami *et al* (2003) evaluated the efficacy and safety of *Acalypha wilkesiana* ointment in superficial fungal skin diseases. Their formulation produced total inhibition of the growth of *Tinea pedis*, *Pityriasis versicolor* and *Candida intetrigo*. A comparative antimicrobial study on two varieties of *Acalypha wilkesiana* (Macrophylla and Hoffmanni) showed that it possessed a broad spectrum of activity on both bacteria and fungi (Oladunmoye, 2006). In a study by Jekayinfa *et al* (1997), the aqueous extract of *Acalypha wilkesiana* (Macrophylla) showed significant antibacterial and antifungal properties *in vitro* and was found to be reasonably useful in the treatment of eczema. The *in vitro* antihelminthic activity of plant extracts of *Acalypha wilkesiana* against *Fasciola gigantica, Taenia solium* and *Pheritimap asthuma* has also been documented (Onocha and Olusanya, 2010).

The objective of this study was to determine and compare the antifungal activities of the extracts from leaves of two cultivars the Macrophylla (cooper leaves) and Hoffmanii (green leaves); fractions and herbal creams from the ethanol extract of *Acalypha wilkesiana* (Macrophylla and Hoffmanni).

## Materials and Methods

### Chemicals

The reagents used for the study include; Sabouraud dextrose agar- SDA (Oxoid, England), Sabouraud dextrose broth- SDB (Oxoid, England), Amphotericin B powder (Sigma- Aldrich), Fluconazole powder (Sigma- Aldrich), Terbinafine powder (Sigma- Aldrich), Dimethylsulphoxide (Sigma- Aldrich). All other reagents were of analytical grade.

### Test organisms

Fungal species used in this study include *Malassezia furfur* ATCC 14521, *Candida albicans* ATCC 10231, *Trichophyton rubrum* ATCC 28188. Clinical isolates of *Microsporum canis* and *Epidermophyton floccosum* were obtained from Department of Microbiology and Biotechnology, National Institute for Pharmaceutical Research and Development, Abuja, Nigeria. The fungal isolate was stored on sabouraud dextrose agar slants at 4 °C until when needed.

### Collection of plant material

The fresh leaves of the two varieties of *A. wilkesiana* were collected from the botanical garden of the National Institute for Pharmaceutical Research and Development (NIPRD), Abuja, Nigeria the 13^th^ of July 2016 (9.0637 ^o^N, 7.3382 ^o^E). Voucher specimens of both varieties of *A. wilkesiana* were deposited in the herbarium at the Department of Medicinal Plant Research and Traditional Medicine, NIPRD, Abuja Nigeria and the plants were given herbarium specimen numbers NIPRD/H/ 6788 for the Macrophylla (copper leaves) and NIPRD/H/ 6789 for Hoffmanii (green leaves). The fresh leaves were separated, shade dried and ground to powder using mortar and pestle.

### Extract Preparation

One kilogram of each variety of the powdered leaves was successively macerated in 5 L of n- hexane, ethyl acetate, ethanol, and water for 48 h sequentially and filtered. After each extraction, the extracts were concentrated, dried, weighed and stored in sterile containers at maximum of 25 °C. The yield and percentage yield were calculated.

### Culture preparation

A loopful of 48 h surface growth of each of the culture (yeast) and spore (dermatophytes) was transferred to 5 mL of 0.9 % NaCl solution (with agitation to disperse spores) and homogenous suspension of it was used for inoculation. The spores and yeast counts (1.0 x 10^6^ cfu/mL) were quantified using a haemocytometer (Sharma *et al.*, 2011; Runyoro *et al.*, 2006).

### Bioassay Guided Fractionation

Column chromatography was used to further separate the solvent fraction with highest antimicrobial activity from serial exhaustive extraction (Masoko *et al.*, 2005). The ethanol fractions from the *A. wilkesiana* were dried in a rotary evaporator. The wet method for packing of chromatographic columns was used; silica gel 60 was made into slurry with n-hexane and then poured slowly into a column (15.5 cm x 10 cm), on top of a small amount of cotton wool. The sample was dissolved in 10 ml of ethanol and the mixed thoroughly with equal weight of silica gel 60 in a mortar and pestle. The mixture was air-dried made into slurry with ethanol and poured neatly on top of the silica in the column. Filter paper cut to the internal diameter of the column and cotton-wool were placed on top of the sample to prevent disturbance at the surface during solvent introduction. The fractions were eluted slowly in the presence of vacuum (reverse pressure) by addition of the solvent (n-hexane (100 %), n-hexane – ethyl acetate (90:10, 80:20, 70:30, 60:40, 50:50, 40:60, 30:70, 20:80), ethyl acetate (100 %), ethyl acetate – ethanol (80:20, 70:30, 60:40, 50:50, 40:60, 30:70) and ethanol (100 %) one at a time. The solvent was allowed to run through the column; until all the solvent had been collected in the beakers through a separating funnel. The beakers were allowed to evaporate overnight under a cool stream of air and separated by developing in a tank mobile phase (n-hexane: ethylacetate: ethanol; 3:2:1).on a thin layer chromatogram consisting of silica gel stationery phase Ultravoilet light was then used to identify the various separated spots on the chromatogram before spraying with sulphuric acid.

### Formulation of vanishing herbal cream from ethanol extracts of *A. wilkesiana*

The ethanol extracts from the leaves of both varieties of *A. wilkesiana* leaves were formulated into cream (Table 1) by fusion method. Simply, sorbitan mono-oleate (Span® 80), white beeswax, liquid paraffin and white soft paraffin where melted together and cooled to 60 °C. Thereafter, the extracts (1, 2, 3, 7.5 and 10 %) were incorporated while mixing thoroughly, poured into a clean container and allowed to cool.

**Table 1:** Formulation of vanishing herbal cream from ethanol extracts of *A. wilkesiana*

Ingredients	Concentration (w/w %)					
	1.0	2.0	3.0	7.5	10.0	Base
Sorbitan mono-oleate (Span® 80)	6.0	6.0	6.0	6.0	6.0	6.0
Bees wax	3.0	3.0	3.0	3.0	3.0	3.0
White soft paraffin	36.0	36.0	36.0	36.0	36.0	36.0
Liquid paraffin	15.0	15.0	15.0	15.0	15.0	15.0
Extract	1.0	2.0	3.0	7.5	10.0	0.0
Purified water	39.0	38.0	37.0	32.5	30.0	40.0

### Antimicrobial screening of extracts and herbal creams

The extracts and herbal creams were screened for their antifungal activity against the test organisms by disc diffusion method (Arora *et al.*, 2013). Sabouraud dextrose agar plates were inoculated with the test isolate by spreading the standardized inoculum on the surface of the agar plate with sterile swab stick. Holes of diameter 6 mm were bored in the inoculated agar plates and 100 L of each of the extract solutions at 16 mg/mL, 8 mg/mL and 4 mg/mL or dissolved creams at concentrations of 3.0, 2.0 and 1.0 % were introduced into the wells. Terbinafine HCl solution and fluconazole discs (10 µg/mL) served as positive control whereas the disc containing 10% dimethylsulphoxide (DMSO) alone was used as a negative control for antifungal assay. All the plates were incubated at 25°C for 48 h. The antifungal activity was assessed by measuring the diameter of the zone of inhibition in millimeters formed from observation of the clear zones surrounding each well. The bioassay was performed in triplicate to calculate the mean value.

### Determination of Minimum Inhibitory Concentration (MIC)

The extracts from the leaves of *A. wilkesiana* were subjected to antifungal sensitivity testing by broth microdilution method (Santhanam *et al.*, 2014). The 96-microtiter well was prepared by dispensing 95 L of Saboraud dextrose broth (SDB) overlaid with 1 mL of olive oil (for *M. furfur*), SDB (other fungi) and left for 15 mins before adding 5 L of the fungi suspension into each well. One hundred microliters (100 L) from the stock solution of extracts was added into the first well and then followed by two-fold serial dilution down the remaining wells. The last row of wells did not contain the extract thus served as organism viability control. Each plate was shaken for 20 seconds and then incubated at 30 °C for 48 h. At the end of the incubation period, the plates were observed visually for the presence or absence of growth. Minimum inhibitory concentration was calculated as the lowest concentration of the antifungal agent showing no turbidity after incubation, where the turbidity was interpreted as visible growth of the fungi. Each test was performed in triplicate.

### Ethical Clearance

Ethical approval (NIPRD/05:03::05-14) was obtained from the Ethical Committee, Department of Pharmacology and Toxiciology, National institute for Pharmaceutical Research and Development.

### Statistical analyses

The diameters of zones of inhibition and minimum inhibitory concentrations of the extracts, fractions and herbal creams was presented as a mean of three values and standard deviation.

## Results

### Extraction of the Leaves of *A. wilkesiana*

The total and percentage yield of the extracts as shown in Table 2. revealed an increase in the extraction yield with increase in the polarity of the extraction solvents. As a result, the ethanol extract yielded the greatest quantities and least polar hexane extracted the least amount.

**Table 2:** Percentage yield of extracts from 100 g of *A. wilkesiana* leaves varieties using various solvents.

Solvents	AWM Yield (g)	Percentage Yield	AWH Yield (g)	Percentage Yield
Hexane	2.966	2.97	1.725	1.73
Ethyl acetate	1.437	1.44	2.237	2.24
Ethanol	3.700	3.70	3.880	3.88

AWH*- A. wilkesiana* Hoffmanii

AWM*- A. wilkesiana* Macrophylla

### Chromatographic Analysis of Ethanol Extract of the Leaves of *A. wilkesiana*

A total of 35 fractions each were collected from the elution of the ethanol extracts of *A. wilkesiana* Macrophylla (AWM) and *A. wilkesiana* Hoffmanii (AWH). Identical fractions based on the separation on thin layer chromatography were combined giving 8 and 7 fractions altogether for AWM and AWH respectively as follows:

### Antifungal Screening

### Susceptibility of the fungi to various solvent extracts of *A. wilkesiana* leaves (Macrophylla and Hoffmanii)

The extracts of both varieties showed some levels of inhibition against all the organisms. However, less inhibition was seen generally with the hexane extracts. *Candida albicans* was the only organism inhibited by the hexane extracts of the 2 varieties of plants. Inhibitory activity of the extracts against *Microsporum canis* was less than for other organisms. The ethanol extracts produced the highest antifungal activities across all organism types, with zones of inhibition diameter in the range of 7.00 ± 0.00 - 24.00 ± 0.00 mm that was comparable to those of the standard drugs used (Table 4).

**Table 3:** Combination of fractions based on thin layer chromatography

AWM Fractions	Combined fractions	AWH Fractions	Combined fractions
R1	1-3	G1	1-4
R2	4-5	G2	5-7
R3	6	G2	8-10
R4	7-11	G4	11-14
R5	12-16	G5	15-21
R6	17-22	G6	22-31
R7	23-30	G7	32-35
R8	31-35		

AWH*- A. wilkesiana* Hoffmanii AWM*- A. wilkesiana* Macrophylla

**Table 4:** Susceptibility of the fungal isolates to different solvent extracts of *A. wilkesiana* leaves (Macrophylla and Hoffmanii) at 16 mg/mL

Organisms	AWM (Zone of Inhibition in mm)			AWH (Zone of Inhibition in mm)			Control (mm)
	HEX	ETA	ET	HEX	ETA	ET	
MF	0.00±0.00	0.00±0.00	18.00±0.00	0.00±0.00	0.00±0.00	17.00±0.00	24.00±0.00
Ca	14.50±0.57	20.00±0.00	15.0±0.00	15.0±0.33	15.00±0.33	18.00±0.57	34.00±0.00
EF	0.00±0.00	0.00±0.00	20.00±0.33	0.00±0.00	0.00±0.00	20.00±0.00	20.00±0.00
TR	0.00±0.00	0.00±0.00	22.00±0.00	0.00±0.00	0.00±0.00	24.00±0.00	20.00±0.00
MC	0.00±0.00	0.00±0.00	8.00±0.00	0.00±0.00	0.00±0.00	7.00±0.00	20.00±0.00

**Keys:** HEX- hexane; ETA- ethyl acetate; ET- ethanol

Control- Terbinafine HCl 10 µg/mL (MF and Ca) and fluconazole discs 10 µg (EF, TR and MC)

Ca 10231- *C*. *albicans* ATCC 2876

MF*- Malassezia furfur* ATCC 14521 TR*- Trichophyton rubrum* ATCC 28188

EF *-Epidermophyton flocossum*

MC*- Microsporum canis AWH- A. wilkesiana* Hoffmanii

*AWM- A. wilkesiana* Macrophylla

### Susceptibility of the test organisms to the ethanol extracts of *A. wilkesiana* leaves (Macrophylla and Hoffmanii)

Concentration-dependent inhibition of zones of inhibition of the test fungi were produced by the extracts. The zones of inhibition for *Trichophyton rubrum* (22.00±0.00, 24.00±0.00) and *Epidermophyton floccosum* (20.00±0.00, 20.00±0.00) were the largest for AWH and AWM respectively while that for *M. canis* (8.00±0.00, 7.00±0.00) was the lowest at a concentration of 16 mg/ml (Table 5.)

**Table 5:** Susceptibility of the test organisms to the ethanol extracts of *A. wilkesiana* leaves (Macrophylla and Hoffmanii)

Organisms	AWM (Zone of Inhibition; mm)			AWH (Zone of Inhibition; mm)			Control (mm)
	16 mg/mL	8 mg/mL	4 mg/mL	16 mg/mL	8 mg/mL	4 mg/mL	
MF	18.00±0.00	15.50±0.57	12.00±0.00	17.00±0.00	14.00±0.33	12.00±0.33	24.00±0.00
Ca	15.0±0.00	15.00±0.33	15.00±0.33	18.00±0.57	17.00±0.33	16.00±0.33	34.00±0.00
MC	8.00±0.00	8.00±0.00	6.00±0.00	7.00±0.00	6.00±0.00	6.00±0.00	20.00±0.00
EF	20.00±0.00	22.00±0.00	16.00±0.00	20.00±0.00	20.00±0.00	10.00±0.00	20.00±0.00
TR	22.00±0.00	18.00±0.00	15.00±0.00	24.00±0.00	14.00±0.00	12.00±0.00	20.00±0.00

Control- Terbinafine HCl 10 µg/mL (MF and Ca) and fluconazole discs 10 µg (EF, TR and MC)

CA-C. *albicans* ATCC 10231 MF*- Malassezia furfur* ATCC 14521

TR- *Trichophyton rubrum* ATCC 28188

EF *-Epidermophyton flocossum*

MC*- Microsporum canis*

*AWH- A. wilkesiana* Hoffmanii

*AWM- A. wilkesiana* Macrophylla

### Minimum inhibitory concentration (MIC) of the ethanol extract and column fractions from ethanol extracts of *A. wilkesiana* leaves (Macrophylla).

Fraction R4 had the best activity overall against the fungal organisms with *Candida albicans* being the most susceptible (156μg/ml).

The greatest activity of the crude extract of AWM was against *T. rubrum* (250 µg/ml); *C. albicans* (250 µg/ml) (Table 6).

**Table 6:** Minimum inhibitory concentration (MIC) of the ethanol extracts and column fractions from ethanol extract of *A. wilkesiana* leaves (Macrophylla)

ORG	Minimum Inhibitory Concentration (µg/mL)									
	AWM	R1	R2	R3	R4	R5	R6	R7	R8	CON
MF	4000.00±0.00	1250.00± 0.33	1250.00± 0.57	1250.00± 0.00	1250.00± 0.33	2500.00± 0.00	1250.00± 0.33	625.00± 0.57	2500.00±0.00	12.50± 0.00
CA	250.00±0.00	1250.00± 0.00	313.00± 0.00	156.00± 0.57	156.00± 0.00	156.00± 0.33	625.00± 0.00	1250.00± 0.00	313.00±0.00	12.50± 0.00
MC	>8000.00±0.00	1250.00± 0.00	2500.00± 0.00	2500.00± 0.33	1250.00± 0.00	1250.00± 0.00	2500.00± 0.00	2500.00± 0.57	>8000.00±0.00	6.25± 0.00
EF	4000.00±0.00	1250.00± 0.00	2500.00± 0.00	2500.00± 0.33	1250.00± 0.33	156.00± 0.00	2500.00± 0.00	2500.00± 0.00	>8000.00±0.00	12.50± 0.00
TR	250.00±0.00	1250.00± 0.00	1250.00± 0.00	2500.00± 0.00	1250.00± 0.00	4000.00± 0.00	2000.00± 0.00	4000.00± 0.00	3000.00± 0.00	12.50± 0.00

Key: CA- C. *albicans* ATCC 10231

MF- *M. furfur* ATCC 14521

TR- *T. rubrum* ATCC 28188

EF -*E. flocossum* MC- *Microsporum canis*

AWM- *A. wilkesiana* Macrophylla

ORG - *Organisms*

CON- control- FCZ- *Fluconazole (MF, CA);*

TER - *Terbinafine (MC,CF and TR)*

*NA- Not applicable*

### Minimum inhibitory concentration (MIC) of the ethanol extracts and column fractions from ethanol extract of *A. wilkesiana* leaves (Hoffmanii)

The lower MICs are highlighted. The fraction G3 (third column fraction from ethanol extract AWH), had the best activity against the fungal organisms followed sequentially by G4. The fractions G3 and G4 were the most active against *C. albicans*. The greatest activity of the crude extract of AWH was against *C. albicans* (125 µg/ml) and *T. rubrum* (500 µg/ml) (Table 7).

**Table 7:** Minimum inhibitory concentration of the ethanol extracts and column fractions from ethanol extract of *A. wilkesiana* leaves (Hoffmanii)

ORG	Minimum Inhibitory Concentration (µg/mL)								
	AWH	G1	G2	G3	G4	G5	G6	G7	CON
MF	2000.00±0.00	1250.00±0.00	625.00± 0.57	2500.00± 0.00	2500.00± 0.33	2500.00± 0.00	2500.00± 0.33	4000.00± 0.57	12.50± 0.00
CA	125.00±0.00	313.00±0.00	313.00± 0.00	156.00± 0.57	156.00± 0.00	156.00± 0.33	625.00± 0.00	1250.00± 0.00	12.50± 0.00
MC	4000.00±0.00	>8000.00±0.00	2500.00± 0.00	1250.00± 0.33	1250.00± 0.00	1250.00± 0.00	1250.00± 0.00	2500.00± 0.57	6.25± 0.00
EF	4000.00±0.00	>8000.00±0.00	2500.00± 0.00	156.00± 0.33	625.00± 0.33	2500.00± 0.00	2500.00± 0.57	2500.00± 0.33	12.50± 0.00
TR	500.00±0.00	5000.00± 0.00	3000.00± 0.00	2500.00± 0.00	2500.00± 0.00	2500.00± 0.00	2000.00± 0.00	8000.00± 0.00	12.50± 0.00

CA - *C. albicans* ATCC 10231

MF- *Malassezier furfur* ATCC 14521

TR- *Trichophyton rubrum* ATCC 2188

EF -*Epidermophyton flocossum*

MC- *Microsporum canis*

AWG- *A. wilkesiana* Hoffmanii

ORG – Organisms

Control- FCZ- Fluconazole (MF and CA) TER – Terbinafine (MC, EF and TR) NA- Not applicable

### Susceptibility and antimicrobial activities of the formulated cream from crude ethanol extracts of AWM and AWH

Table 8 demonstrates antifungal susceptibility to the formulated cream of the ethanolic extract of both cultivars. At 1 %w/w concentration, activity was minimal except against *Microsporum canis*, where some inhibition which was absent for other organisms were observed. With higher concentrations up to 3 %w/w, the activity improved but not as high as the activity of the crude ethanol extract.

**Table 8:** Susceptibility of the fungal isolates to the cream formulated with the crude ethanol extracts of *A. wilkesiana* leaves (Macrophylla and Hoffmanii)

Organisms	AWM (Zone of Inhibition ; mm)			AWH (Zone of Inhibition ; mm)			

	3 %w/w	2 %w/w	1 %w/w	3 %w/w	2 %w/w	1 %w/w	Control
MF	8.00±0.00	5.50±0.57	0.00±0.00	7.00±0.00	4.00±0.33	0.00±0.00	0.00±0.00
CA	5.00±0.00	5.00±0.33	0.00±0.00	8.00±0.57	7.00±0.33	0.00±0.00	0.00±0.00
MC	6.00±0.00	5.00±0.00	4.00±0.00	5.00±0.00	5.00±0.00	4.00±0.00	0.00±0.00
EF	10.00±0.00	8.00±0.00	0.00±0.00	10.00±0.00	6.00±0.00	0.00±0.00	0.00±0.00
TR	12.00±0.00	8.00±0.00	0.00±0.00	14.00±0.00	4.00±0.00	0.00±0.00	0.00±0.00

CA- C. *albicans* ATCC 10231

MF- M. *furfur* ATCC 14521

TR- *T. rubrum* ATCC 28188

EF -*E. flocossum*

MC- *M. canis*

AWH- *A. wilkesiana* Hoffmanii

*AWM- A. wilkesiana* Macrophylla.

Control- FCZ- Fluconazole disc 10 µg (MF and CA)

TER - Terbinafine disc 10 µg (MC, EF and TR)

### Minimum Inhibitory Concentration (MIC) determination of 7.5 %w/w and 10 %w/w cream formulations of the ethanol extracts of *A. wilkesiana* leaves (Macrophylla and Hoffmanii)

The cream formulated from higher concentrations of the extract produced good antifungal activity. Creams from both varieties of the plant were most active against *C. albicans* while AWH was more than AWM active against *M. furfur and T. rubrum C. M. canis* was the least susceptible and comparable to the negative controls (Table 9).

**Table 9:** Minimum Inhibitory concentration determination of cream formulated with the ethanol extracts of *A. wilkesiana* leaves (Macrophylla and Hoffmanii).

ORG	AWM (MIC; µg/mL)		AWH (MIC; µg/mL)		Blank Control	cream
	10 %w/w	7.5 %w/w	10 %w/w	7.5 %w/w	0 %w/w	1 %w/w
TR	1250.00±0.00	2500.00±0.57	625.00±0.00	2500.00±0.33	>8000.00±0.00	16.00±0.00
MC	>8000.00±0.00	>8000.00±0.00	>8000.00±0.00	>8000.00±0.00	>8000.00±0.00	16.00±0.00
MF	1250.00±0.00	2500.00±0.57	625.00±0.00	1250.00±0.33	>8000.00±0.00	16.00±0.00
CA	312.50±0.00	625.00±0.57	312.50±0.00	625.00±0.33	>8000.00±0.00	8.00±0.00

CA- C. *albicans* ATCC 10231

MF- *M. furfur* ATCC 14521

TR- *T. rubrum*

EF - *E. flocossum*

MC- *M. canis*

*AWH- A. wilkesiana* Hoffmanii

*AWM- A. wilkesiana* Macrophylla

Control- FCZ- Fluconazole (MF and CA)

TER - Terbinafine (MC, EF and TR)

## Discussion

### Antifungal activity of extracts of *Acalypha wilkesiana* (Macrophylla and Hoffmanii)

Both plant varieties demonstrated antifungal activity against all the test fungi except *Microsporum canis*. Overall, *C. albicans* and *T. rubrum* were the most susceptible while *C. albicans* and *M. furfur* showed greater susceptibility to AWH than AWM.

The strong inhibition and broad spectrum (yeast and dermatophytes) activity displayed by the ethanol extract as compared to the ethyl acetate and hexane extracts could be linked to the ability of this solvent to extract more anti-fungal components of the plants than the other solvents. According to Zohra *et al.*, 2011, the phytoconstituents present in plants possess varying degrees of solubility in different solvents, which is due to the different classes of constituents present in the plant and the polarities of the solvents (Santhanam *et al.*, 2014). In a study by Egwin and Yakubu, 2017 it was reported that most phytoconstituents with antimicrobial activities like tannins, saponins and triterpenes from *A|. wilkesiana* leaves were best extracted by ethanol and least by pet ether as these phytoconstituents were more soluble in polar solvents.

In a study of 6 medicinal plants including *A. wilkesiana* done in Kinshasa (Cimanga *et al.*, 2014), the aqueous extract of *A. wilkesiana* (Macrophylla) had the best activity against *C. albicans* (MIC -31.2µg/ml) among a spectrum of bacteria and fungi, this agrees with the result of our study which revealed that *C. albicans* was the most susceptible fungi to the crude ethanol extract of *A. wilkesiana*. Ezekiel *et al*, 2009 in another study found the methanol extract of the plant to be less active against *C. albicans* than other organisms. However, the cultivar of *A. wilkesiana* studied was Macfeeana, which is different from the 2 variants studied.

Although dermatophytic infections are common, few studies have looked at the spectrum of activity of this plant on the common causative agents. This study looked at 3 types of dermatophytes; *E. floccusum, T. rubrum* and *Microsporum canis*. The activity of the crude extract against *T. rubrum* was the best, while the R5 and G3 fractions were more effective than the crude extract against *E. flocossum* and *Microsporum cani*s. However, susceptibility of *M. canis* to the crude extract was not as pronounced as the other dermatophytes. This may account for the 54 % and 100 % cure rate seen in patients with *Tinea corporis* and *T. pedis* respectively in a clinical study by Oyelami *et al.*, (2003). This would suggest that some forms of dermatophytoses might not be as easily treated with the plant except perhaps in higher concentrations and / or longer duration of administration. Haruna *et al*. (2013) found *C. albicans* to be more susceptible to *A. wilkesiana* than *T. mentagrophytes* (Haruna *et al.*, 2013), which also tallied with findings by Alade *et al*. (1993), showing better antifungal activity based on MIC (Alade and Irobi, 1993). Susceptibility was best with *T. rubrum, T. mentagrophytes, C. albicans* in that study with *T. rubrum* and *T. mentagrophytes* having lowest MICs. Majekodunmi and Nubani (2014), reported best activity with *C. albicans* (Majekodunmi and Nubani, 2014).

Good antifungal activity of both variants of the plant is confirmed in this study with R4 and G3 fractions producing better effect than the crude extracts and the crude extract of Hoffmanii cultivar (AWH) showing better activity against the test fungi than the Macrophylla (AWM). This is the first *in-vitro* study of the antifungal effect of *A. wilkesiana* on *Malassezia furfur* to the authors’ knowledge. Though it’s curative effect on clinical lesions of *Pityriasis versicolor* has been recorded before (Azubuike *et al.*, 2013). This is important because the traditional healers have used this plant for ages to treat pityriasis versicolor and seborrheic dermatitis, diseases with aetiological link to *M. furfur*.

### Antifungal activity of the herbal vanishing cream of *Acalypha wilkesiana*

Vanishing cream is an oil-in-water emulsion which disappears easily when rubbed on the skin, making it cosmetically acceptable. Vanishing cream has better diffusion qualities because of its thinness, apart from cosmetic acceptability. Considering that this plant is used traditionally for skin infections, the spectrum of activity of the topical preparation of the plant was studied. Significant activity was seen with the herbal vanishing cream at lower concentration of 3%w/w, however activity was not as good as the crude extract. The cream had to be constituted with higher concentrations (7.5 %w/w and 10 %w/w) of the extract to achieve similar susceptibility profile to the crude extract. The 10 %w/w cream was observed to have better activity than the crude against some organisms like *C. albicans*, *T. rubrum* and *M. furfur*. However, the best activity was seen with *C. albicans* while the activity of the herbal cream against *M. canis* was poor.

Azuibike *et al*, (2013) demonstrated good antimicrobial activity of 10 % w/w simple and emulsifying ointments of ethanolic extracts of *A. wilkesiana* against *C. albicans*. The activity of the herbal cream was comparable to Funbact A cream® (combination of neomycin and clotrimazole) used as positive control as opposed to that of the simple ointment. However, unlike the findings in our study, the activity of those creams at higher concentrations were however lower than that of the crude ethanolic extracts (Azubuike *et al.*, 2013).

In another study, formulations of the ethanolic extract of the plant in cetagromacol cream, zinc cream and emulsifying wax ointment were active against *Candida albicans* (Sharma *et al.*, 2011). The emulsifying wax preparation had lower antimicrobial activity compared to the other 2 creams. The cetomagrocol cream on the other hand was most active against *C. albicans*. The paraffin in the emulsifying wax may have impeded diffusion leading to a lower activity.

Oladunmoye *et al*, (2006) documented better antimicrobial activity of ethanol extracts of AWM compared to AWH with respect to fungi studied. This is, however, not the case in this study as AWH had better activity against fungal organisms generally particularly *Candida albicans* and *Malassezia furfur*. Interestingly, their study also revealed that, the MIC for green Hoffmanii was lower against *Trichophyton interdigistale*, suggesting a better activity than the Macrophylla variety of the plant.

For most of the organisms, the spectrum of activity of AWM and AWH was similar. *Trichophyton rubrum* seemed to more susceptible to the AWM while AWH was more effective against *Candida albicans* and *Malassezia furfur*. This is important when deciding the variant of the plant to develop for specific organisms.

## Conclusion

The two cultivars of *Acalypha wilkesiana* (Macrophylla and Hoffmanii) have shown to be effective against most of the tested fungi. The ethanolic extract was the most active against the organisms with its best activity against *Trichophyton rubrum* and *Candida albicans*. The activity against *Malassezia furfur* supports its use for *Pityriasis vesicolor* and seborrheic dermatitis. The herbal vanishing cream prepared from the plant extract though less effective than the crude ethanol extract, appears to be promising. This suggests that clinical studies on the topical use of this plant in skin infections should be carried out and further research is important to develop lead compounds that may be useful even in multidrug resistant infections.

### Conflict of Interest

The authors declare that there is no conflict of interest associated with this study.

**Abbreviations:**
AW –: *Acalypha wilkesiana*
AWM-: *Acalypha wilkesiana* Macrophylla
AWH-: *Acalypha wilkesiana* Hoffmanii
PV -: *Pityriasis versicolor*
SDA-: Sabouraud dextrose agar
SDB**-**: Sabouraud dextrose broth
