# ANTI-FUNGAL ACTIVITY OF *ACALYPHA WILKESIANA*: A PRELIMINARY STUDY OF FUNGAL ISOLATES OF CLINICAL SIGNIFICANCE

**DOI:** 10.21010/Ajidv17i1.7

**Published:** 2022-12-22

**Authors:** Katibi Oludolapo Sherifat., Aboh Mercy Itohan, Salawu Oluwakayinsola Adeola, Kola-Mustapha Adeola, Olatunji Lawrence Aderemi

**Affiliations:** 1 Dermatology Unit, Department of Paediatrics and Child health, University of Ilorin, Ilorin, Nigeria; 2 Department of Microbiology and Biotechnology, National Institute for Pharmaceutical Research & Development, Abuja, Nigeria; 3 Department of Pharmacology & Toxicology, National Institute for Pharmaceutical Research & Development, Abuja, Nigeria; 4 Department of Pharmaceutics and Pharmaceutical Microbiology, Faculty of Pharmaceutical sciences, University of Ilorin, Ilorin, Nigeria; 5 Department of Physiology, College of health Sciences, University of Ilorin, Ilorin, Nigeria

In the version of this article initially published, there were errors resulting from merged authors’ names. The following sentences highlight the errors and the corrected details:

**KATIBI Oludolapo Sherifat.^1^, ABOH Mercy Itohan^2^ SALAWU Oluwakayinsola Adeola, KOLA-MUSTAPHA Adeola^4^ , OLATUNJI Lawrence Aderemi^5^**

